# A Study on Generative Models for Visual Recognition of Unknown Scenes Using a Textual Description

**DOI:** 10.3390/s23218757

**Published:** 2023-10-27

**Authors:** Jose Martinez-Carranza, Delia Irazú Hernández-Farías, Victoria Eugenia Vazquez-Meza, Leticia Oyuki Rojas-Perez, Aldrich Alfredo Cabrera-Ponce

**Affiliations:** 1 Department of Computational Science, Instituto Nacional de Astrofisica, Optica y Electronica (INAOE), Puebla 72840, Mexico; dirazuhf@inaoep.mx (D.I.H.-F.); victoria.vazquez@inaoep.mx (V.E.V.-M.); oyukirojas@inaoep.mx (L.O.R.-P.); 2 Faculty of Computer Science, Benemerita Universidad Autonoma de Puebla (BUAP), Puebla 72570, Mexico; aldrich.cabrera@alumno.buap.mx

**Keywords:** visual scene recognition, generative models, textual descriptions, diffusion model, CLIP, visualBERT

## Abstract

In this study, we investigate the application of generative models to assist artificial agents, such as delivery drones or service robots, in visualising unfamiliar destinations solely based on textual descriptions. We explore the use of generative models, such as Stable Diffusion, and embedding representations, such as CLIP and VisualBERT, to compare generated images obtained from textual descriptions of target scenes with images of those scenes. Our research encompasses three key strategies: image generation, text generation, and text enhancement, the latter involving tools such as ChatGPT to create concise textual descriptions for evaluation. The findings of this study contribute to an understanding of the impact of combining generative tools with multi-modal embedding representations to enhance the artificial agent’s ability to recognise unknown scenes. Consequently, we assert that this research holds broad applications, particularly in drone parcel delivery, where an aerial robot can employ text descriptions to identify a destination. Furthermore, this concept can also be applied to other service robots tasked with delivering to unfamiliar locations, relying exclusively on user-provided textual descriptions.

## 1. Introduction

The parcel delivery industry has grown significantly in recent years due to the popularisation of e-commerce and online shopping. However, there are regions around the world where inadequate urban planning and maintenance make delivery a daunting task. Even when a courier has the delivery location’s address and the GPS coordinates on a map, they often encounter challenges in locating the exact delivery destination, especially if the courier is unfamiliar with the neighbourhood. This is known as the last-mile delivery problem [1], a metaphor that illustrates that the last part of the delivery trip, defined between the local warehouse and the final destination (usually located in the same region/city/town as the warehouse), is the most expensive and time-consuming stage [2].

In anticipation of any location-finding issues, several companies request a textual description of the target destination. This may include the appearance of buildings and distinctive landmarks such as trees, cars, lampposts, or any other feature that helps to recognise the target location. Humans have the ability to read this textual description and “imagine” what the target destination would look like. For instance, if the description indicates that a red car is parked in front of the target destination, which at the same time has a palm tree placed in the front yard, a human is capable of imagining these objects with no particular visual features but rather general semantic characteristics. There are thousands of car shapes and numerous variations of the colour red. Nevertheless, humans have a general understanding of the concept of a car and can recognise the colour red, regardless of its shade or gradient. Even if the person has never seen the car before, the semantic attributes are enough for the human to identify it. In the event that more than two cars were found in the scene, the second object, the palm tree, would help to disambiguate the target location, but again, no detailed description of the palm is required. The spatial relation between the car and the tree becomes more useful than precisely detailed information of the target location and the surrounding objects.

This human capability of being able to “imagine” a place from a textual description is what motivates this work. The primary question is whether a computational procedure can be implemented such that given a textual description, the computer can “imagine” a visual representation of such description. We are aware that the word “imagine” is broad and may be difficult to capture into a computational procedure. However, the last couple of years have seen the emergence of novel techniques known as generative models [3], where given a text, a computer is capable of generating an image [4,5]. Therefore, a second question is whether these generative models could be enough to “recognise” a scene where one has not been before.

To answer the aforementioned question, this paper provides preliminary insights into the use of generative models, which are used to generate an image from a textual description (e.g., diffusion models such as Stable Diffusion [6]) or a text generated from a target image (known as image captioning [7]). Given a set of textual descriptions (provided by humans) of target places and generated images from these descriptions, what are the options to compare them against a given target image? To answer this question, we explore the use of embedding representations such as Contrastive Language–Image Pre-training (CLIP) [8], studied in our previous work [9], but now complemented with the study of another embedding method known as VisualBERT [10], which also provides a numerical representation of an image.

**Figure 1:**
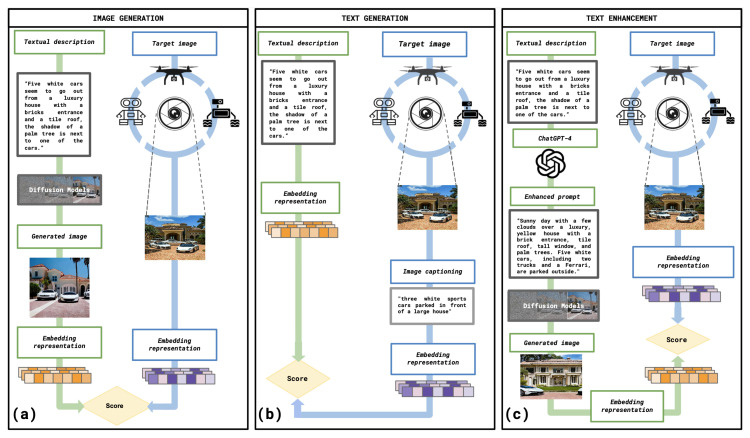
(**a**) Stable Diffusion model [6] to generate an image from the provided textual description. (**b**) Image captioning [7] to generate a caption compared to the original textual description. (**c**) User’s textual description enhancement using ChatGPT-4 [11].

In this manner, we explore three possible strategies (see also Figure 1):

- Image generation: We generate an image given the textual description provided by the user and compare it against the target image.
- Text generation: We compare the textual input against text generated from image captioning of the target image.
- Text enhancement: We also delve into the use of tools such as ChatGPT [11] to perform prompt engineering seeking to “generate” a more concise textual description of a target location and evaluate its impact on the diffusion model.

Our experimental framework shows interesting insights. First, methods such as CLIP and VisualBERT measure different levels of semantic similarity. The former scores better when a prominent object dominates the scene, whereas the latter considers the spatial relationship between objects and their background. Second, a direct comparison between textual descriptions provided by humans and generated text (captioning) may not be enough and seems to be less effective when compared to a visual comparison. Third, tools such as ChatGPT are an option to engineer the original textual description input by the user, and in such cases, our results are useful to assess whether such an enhanced description has an impact on the generated image or not.

Therefore, the results obtained in this study contribute to understanding, both qualitatively and quantitatively, the impact of employing generative tools in conjunction with state-of-the-art multi-modal numerical representations, specifically CLIP and VisualBERT embeddings, in order to address the problem of visual recognition of unknown scenes.

There are several applications that could benefit from this research; the first and foremost is that of parcel delivery using drones, where an aerial robot could use the textual description to recognise the target place in the case when the address or GPS location is not enough. Nevertheless, this could be extended to any other service robot that has to deliver a package to a place where it has never been before and for which a textual description is the only aid provided by the user.

This paper has been organised as follows to convey our approach. Section 2 discusses the related work; Section 3 describes our approach in more detail; Section 4 presents our experimental framework; and finally, Section 5 outlines our conclusions and future work.

## 2. Related Work

With the advent of deep learning in the last years, the progress achieved by different AI areas as its own has been remarkable. Recently, many efforts have been made to develop multimodal approaches using different information modalities: visual, textual, speech, etc. In particular, there is one combining computer vision and natural language processing capabilities to address very challenging tasks such as (i) image captioning, aiming to generate a textual description from an input image [7]; (ii) visual question-answering, which aims to find answers by means of a question in natural language and a related image [12]; (iii) image retrieval, the objective of which is to retrieve the data in a given modality by the cues provided in another modality [13], i.e., by providing an input text, the system must retrieve relevant images and vice versa; (iv) phrase grounding, involving object detection from an input image and a phrase in natural language [14]; and (v) image generation, which aims to generate an image from the information provided by a textual description in natural language [15].

Generating an image from a textual input is a challenging task that has been addressed from different perspectives. One of the pioneer proposals is alignDRAW [16], which generates images by an iterative process incorporating the use of textual description by means of a soft attention mechanism. More recently, image generation has been addressed by means of generative adversarial networks [17] and Transformer-based architectures [18]. Another alternative is the diffusion models inspired using non-equilibrium thermodynamics [19] that have outperformed the state-of-the-art. Very powerful image generation models have been made publicly available online through simple interfaces, allowing people beyond the research community to use them in a wide range of applications. Among them are *Imagen* [20] and *DALLE-2* [21]. Furthermore, there is *Stable Diffusion* [6] the source code and model weights of which are available for those interested in fine-tuning models for downstream tasks.

Apart from generative models, there are other vision–language models capable of performing more general tasks, including comparisons between images and texts, such as CLIP and VisualBERT [10].

The former is trained with a contrastive learning approach using image–text pairs, while the latter comprises a joint contextualised representation of vision and language to capture the semantics between these modalities.

Different artificial intelligence areas can benefit from using vision–language models [22], for example, robotics, where providing a robot with information coming from language and vision could improve its understanding of the environment where it must perform. In spite of its potential applications, the literature on the use of vision, language, and robotics is scarce. As an attempt to improve navigation in 3D environments, Vision-and-Language Navigation [23] provides communication between humans and agents; another proposal is Text2Pos [24], which performs city-scale position location by means of a textual description, but as with most state-of-the-art methods, it also relies on the ability to only recognise previously known areas. Monocular depth estimation methods have taken advantage of language by means of CLIP [25] and by combining object recognition with spoken language [26]. Large language models have also been evaluated as a tool for decision making in autonomous vehicles [27]. DALL-E-Bot [5] is an autonomous robot that exploits DALL-E for rearranging objects in a scene by inferring a textual description.

State-of-the-art visual localisation methods struggle to match visual data with significantly different appearances [28]. To address this issue, some methods attempt to incorporate different place recognition techniques. Real-world navigation tasks must face challenges such as (i) changes in visual appearances due to temporal variations, (ii) diverse viewpoints of the same areas, and (iii) visiting unknown areas, which can impact efficiency and robustness when applied in real-world scenarios [29]. Many existing methods make use of a reference image for recognising the objects or scenes in the explored environment, assuming that the system has an accurate estimation of its position [28].

Providing drones, service robots, or any other autonomous agent with the ability to recognise unknown scenes by means of generative models has not been yet investigated in depth. In this paper, we propose to investigate the use of diffusion models for generating images from a textual description to be compared with a target image that, in a real scenario, could be captured with an onboard camera. In this sense, we assess different strategies regarding the use of automatically generated images and textual descriptions against a target image and human-generated texts.

## 3. Methodology

We depart from the scenario where we have a textual description, provided by a user, of a target image representing an unknown scene. We investigate the application of generative models and embedding representations (e.g., CLIP [8] and VisualBERT [10]) to facilitate the visual recognition of a target image or scene, potentially obtained with a camera mounted on a robotic system such as a drone or a service robot, based on a textual description provided by a user. Exploring the use of generative models, we consider three different strategies, as outlined in Figure 1. In Figure 1a, we employ a Stable Diffusion model [6] to generate an image from the provided textual description, which is then compared to the target image. In Figure 1b, we utilise image captioning [7] to generate a caption based on the target image, which is subsequently compared to the original textual description. In Figure 1c, we enhance the user’s textual description using ChatGPT [11] and assess whether this enhancement improves image generation using the Stable Diffusion model.

The main blocks used in these strategies are a pre-trained generative model that generates an image from text known as Stable Diffusion [6]; image and text embeddings, obtained with CLIP [8] and VisualBERT [10], a pre-trained image captioning model [7] that produces text from an image; and finally, ChatGPT-4 [11] to produce an enhanced version of the textual descriptions provided by the user.

### 3.1. Image Embeddings for Visual Comparison

For the first and third strategies (image generation and text enhancement), we use the diffusion model to generate an image from the textual description. Thus, for the generated image and the target image, we can compute a numerical embedding vector using either CLIP or VisualBERT. These embeddings can be compared using a similarity score based on the cosine distance (with values between −100 and 100). Assuming we have an embedding for a target image $e_{t}$ and one for the image generated with Stable Diffusion $e_{s}$, the similarity score is: 

(1) $$score=100.0\times \cos\left(e_{t},e_{s}\right)=100.0\times \frac{e_{t}\cdot e_{s}}{∥e_{t}∥∥e_{s}∥}$$

Note that CLIP can also be used to generate an embedding from the textual description. Thus, it can be compared against the embedding of the target image. We evaluated this strategy in our previous work [9] and found that the similarity score between a textual description and a target image reaches an average of 30% in the similarity score range of 0 to 100. In contrast, two CLIP embeddings of the same image achieve a score of 100, and if the image begins to change in appearance, the less similar they become; thus, the smaller the score becomes with a tendency towards zero. We also noted that different textual descriptions with significant changes for the same target image produce scores with no significant difference. This would make it difficult to assess whether one image corresponds better to a textual description than another. Therefore, we argue that working in the visual space provides more discriminative information whose similarity with the target image can be reflected in the score. In this work, textual and image embeddings are numerical vectors of 512 float numbers.

The VisualBERT model was specifically designed to capture the rich semantics present in both images and their associated textual descriptions. This is facilitated by the intricate interplay between words and regions within object proposals, enabling the model to grasp the complex associations between text and images. VisualBERT operates with two primary objectives: predicting masked words based on the visual context and the provided text and determining whether the provided text corresponds accurately to the image. Note that VisualBERT cannot be used to directly compare two texts, as with CLIP.

VisualBERT encompasses different tasks, such as visual question answering (VQA), visual commonsense reasoning (VCR), and natural language for visual reasoning (NLVR). We opted to explore VQA where the model responds to a textual question with a textual answer, effectively limiting its output to specific queries. This approach excludes general details and precludes the utilisation of the image’s direct visual characteristics. Hence, we selected the VisualBERT model pretrained on the COCO dataset for our purposes.

To evaluate the generated images, akin to the process in CLIP, VisualBERT requires both an image and a textual description as input to generate an embedding representation. In Section 4, we will indicate what textual description was fed to VisualBERT in order to generate the corresponding embedding vector. The latter, encapsulating the visual and textual information of the image, was obtained by extracting the output from the last hidden state, resulting in a 768-dimensional embedding. The similarity score between the target image and the generated image was also achieved using the cosine similarity outlined in Equation (1), as in the CLIP evaluation methodology.

### 3.2. Textual Comparison via Image Captioning

As a second evaluation strategy, we decided to consider only textual information. In this case, human-generated textual descriptions of a given scene were compared with a corresponding automatic description obtained by means of a pre-trained image captioning model applied over each target image. Then, these automatic descriptions could be denoted as “target texts”. Therefore, the task was to determine which of the human-generated textual descriptions is the most similar to the target text. For text representation, we exploited three different methods without applying any kind of pre-processing:

- CLIP. Since it allowed us to generate both textual and image based embeddings representations, we extracted the embeddings of the textual descriptions. It is important to mention that CLIP has a constraint regarding the maximum length of the textual inputs, which cannot exceed 77 tokens. Thus, when a textual description is longer than 77 tokens, it is truncated.
- GloVe. By using a pre-trained word embedding model called GloVe [30], we calculated a sentence embedding representation by calculating an average vector of all the words contained in the textual descriptions.
- SentenceBERT. We used a Transformer-based model especially suited for semantic similarity comparison between two sentences denoted as SentenceBERT [31]. This model allowed us to encode each textual description as a single embedding.

In this text-to-text comparison, once we have embedding-based representations it is also possible to use the cosine similarity as previously defined.

### 3.3. Enhanced Textual Description with ChatGPT

As will be described further in our experimental framework, we requested users to provide more than one textual description of a target image, seeking to obtain variation in the generated images. As one could imagine, an image can be described in many different manners, and the level of detail could vary from user to user.

Given the variation among the different textual descriptions of the same target image, we decided to use ChatGPT to produce an enhanced version of the textual descriptions. First, we calculated the average number of words for all the textual descriptions of a target image. As a prompt for ChatGPT, we provided the textual descriptions of a target image plus the following text:

*“Given these descriptions, could you mix them to generate the best description whose number of words is around N words?”*

In the prompt above, *N* is the average number of words in the textual descriptions. The generated textual description was passed to Stable Diffusion to generate a new image that could be compared against the target image. If textual descriptions are seen as prompts for the Stable Diffusion model, this strategy aims to provide what we call an enhanced textual description that could potentially generate a better image that could be compared against the target image, this is, an image with more useful semantic information. Section 4.3 discusses our findings regarding this strategy.

## 4. Experimental Framework

We depart from the fact that we have target images depicting different outdoor scenes. We asked subjects to provide a textual description of a target image by only looking at it. The participants had not previously viewed the images. They were instructed to focus on the image and then provide a written description when prompted.

We defined our experimental test bench as follows. We selected 5 images from the internet without seeking a particular appearance rather than the images that should correspond to an outdoor scene. This was motivated by the delivery scenario, where a courier typically is looking for an outdoor destination. These images were: (1) a house with cars parked in front of it; (2) a food truck selling hot dogs; (3) a kiosk; (4) a basketball court; and (5) a house with a swimming pool. Three subjects were requested to view these images and provide textual descriptions of the scenes. To evaluate variations in the output of our methodology, we asked participants to provide 10 textual descriptions of each target image.

### 4.1. Image Generation from User’s Textual Descriptions

We used Stable Diffusion as an image generation model, which requires a prompt in the form of a text describing the image to be generated. Hence, we used the subjects’ textual descriptions as prompts. The Stable Diffusion model was set to generate 10 images per prompt. This means a total of 100 images were generated per target image. Once the images were generated, we used CLIP and VisualBERT to obtain the corresponding embedding vectors. Beforehand, we also computed the embedding vectors of the 5 target images.

However, before using VisualBERT, its textual input must be converted into an appropriate format using a BERT Tokeniser. In our case, we utilised the BERT Base Uncased model [32], which translates each token in the input sentence into its corresponding unique IDs. Furthermore, the VisualBERT model lacks a built-in function to retrieve the generated visual embeddings from an image. Consequently, we implemented Detectron2 from Facebook AI [33], drawing inspiration from the fundamental methods outlined by the authors of VisualBERT. Additionally, we changed the chosen pre-trained model for mask R-CNN X-101-32x8d FPN [34], selected for having the best accuracy in mask detection performance against other pre-trained models for the R-CNN mask model (this enhanced the final scores obtained from the similarity evaluation).

Detectron2 implementation serves to provide the regional detection and segmentation necessary for entity identification. Moreover, Detectron2 gives the chance to manipulate the number of masks over the image. Therefore, we conducted experiments aimed at controlling the number of masks generated over the images, but finally, we decided to set a minimum of 10 and a maximum of 100 masks per image to keep the model as faithful as possible to the VisualBERT authors’ original implementation. The experiments performed also demonstrated the importance of the number of masks on the image to recognise regions of interest. This exploration revealed the significance of mask quantity and textual description; mask quantity impacts the number allowed over the image and the resultant score. Additionally, the textual description directly affects the score.

Therefore, we computed the embeddings for the 5 target images using CLIP and VisualBERT. For the latter, we fed VisualBERT with the target image together with its corresponding automatically generated textual descriptionby means of a pre-trained image captioning model of BLIP [35]. As for the generated images, CLIP was used seamlessly to generate the embedding vector. However, for VisualBERT, we furnished the model with each image generated by the diffusion model and its corresponding human-generated textual description. Once all embedding vectors were obtained, as explained in Section 3, we used the cosine distance to measure their similarity.

**Figure 2:**
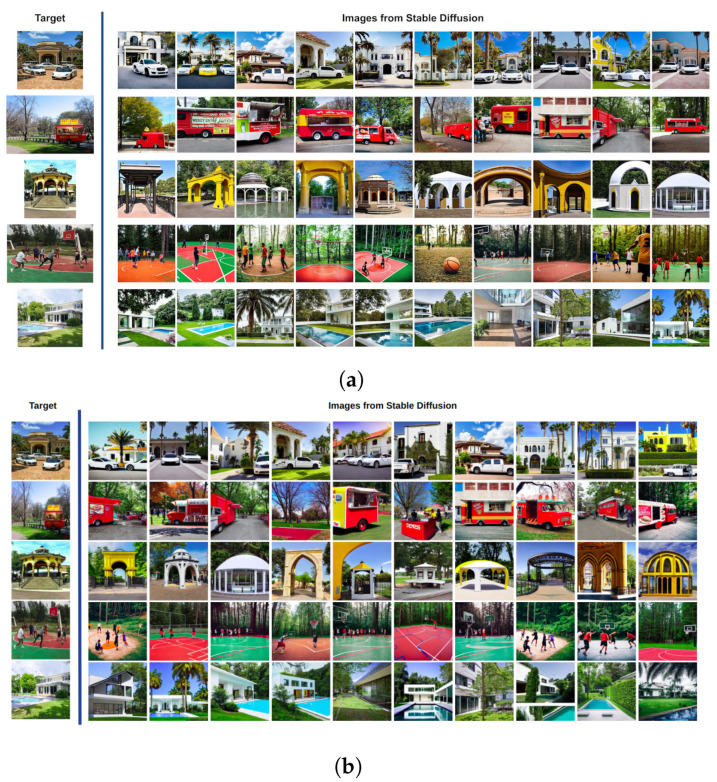
Highest-rated images, ranked by CLIP (**a**) and VisualBERT (**b**) scores using cosine distance (Equation (1)). Each column features the highest-scoring image from prompts by Subject 1. Rows correspond to prompts one through ten.

**Figure 3:**
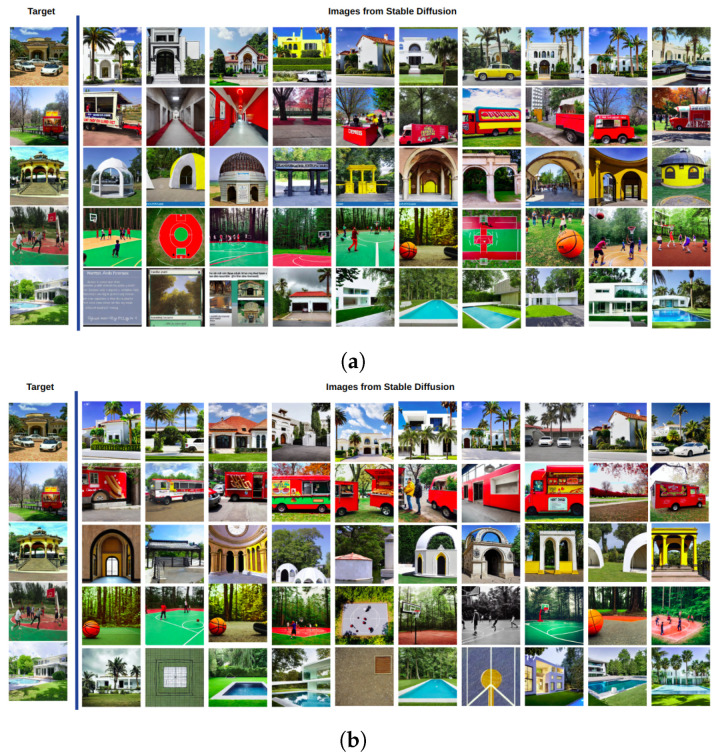
In contrast to Figure 2, we show the lowest-rated images, ranked by CLIP (**a**) and VisualBERT (**b**) scores using cosine distance as well.

Figure 2 shows a mosaic of the best images generated with Stable Diffusion according to the cosine similarity when using the CLIP (Figure 3a) and VisualBERT (Figure 3b) embeddings for Subject 1. Each column corresponds to the best image out of the 10 images generated from prompt 1. Similarly, the second column is the best obtained from prompt 2, and so on until the 10th prompt provided by Subject 1 for each of the target images. Note that in both cases, CLIP and VisualBERT help to draw a set of very similar generated images compared to the target images. In semantic terms, the types of objects, shapes, colours, and backgrounds are very similar. For the sake of comparison, in Figure 3, we also show the worst images generated with Stable Diffusion according to the scores measured with CLIP and VisualBERT. Note that these images contain similar objects to those found in the target images. However, at first sight, the number may be less similar, e.g., the number of cars or persons and the visual appearance of facades, buildings, and roads is also dissimilar (or distorted), and certain objects may also vary. Therefore, this shows that the generative model does not always get it right.

**Figure 4:**
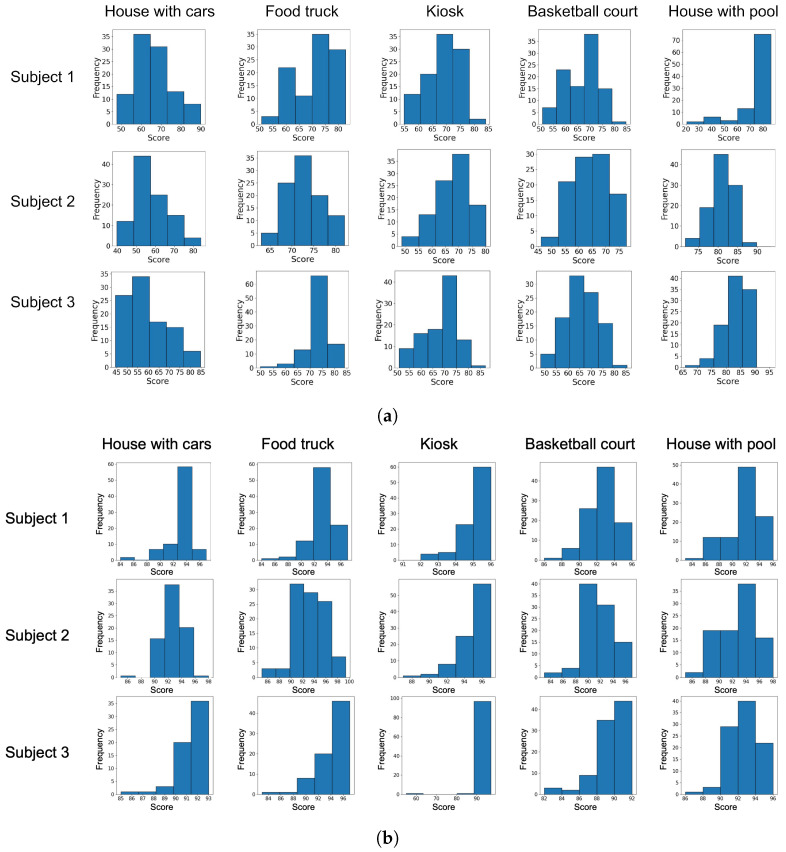
Histogram distribution, per subject, of the scores obtained with CLIP and VisualBERT measuring the similarity between the generated images and the target images. (**a**) Score distribution using CLIP. (**b**) Score distribution using VisualBERT.

Corresponding mosaics for Subjects 1 and 2 are not shown to avoid being repetitive, but similar results were obtained. Instead, to obtain a better picture of the score distribution for both embeddings and for each participant, Figure 4 shows the score distribution per participant and for each target image. The first thing to highlight is that the distribution for VisualBERT (Figure 4b) tends to accumulate towards the right, closer to 100, and with more frequently higher score values than those obtained with CLIP (Figure 4a).

Keep in mind that we do not intend to directly compare CLIP against VisualBERT in the score scale, as these models were trained with a different methodology and datasets. However, the histograms help to show that the generated image scores vary in terms of visual similarity, measured through one embedding or another, but within such a distribution, there is a set of images that could be very similar to the target images.

**Figure 5:**
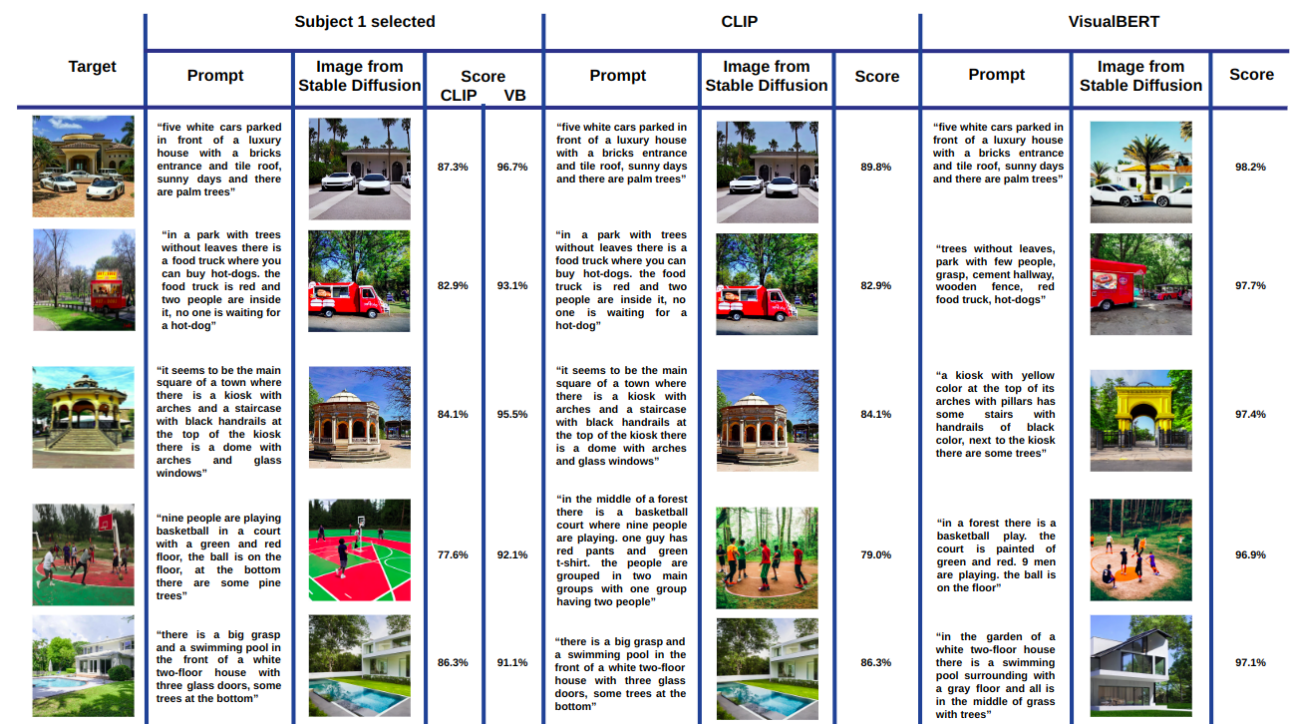
Images most similar to the target image from Subject 1 prompts through three criteria: The one chosen by the subject (selected), the one with the highest CLIP score, and the one with the highest VisualBERT score.

Taking inspiration from previous research that evaluated the effectiveness of generative models through human assessments [36], we also had all participants select the image they believed was the closest match to the target image. In this manner, it is possible to compare them against those images with the highest score obtained with CLIP and VisualBERT. This comparison is shown in Figure 5. The first column shows each target image. The second column headed as “Subject 1 selected” shows the image selected by Subject 1 and the prompt from which it was generated. Scores obtained with CLIP and VisualBERT embeddings are also shown. The second and third columns show the best image according to the score evaluated with CLIP and with VisualBERT, as much as the prompt from which these images were obtained.

**Figure 6:**
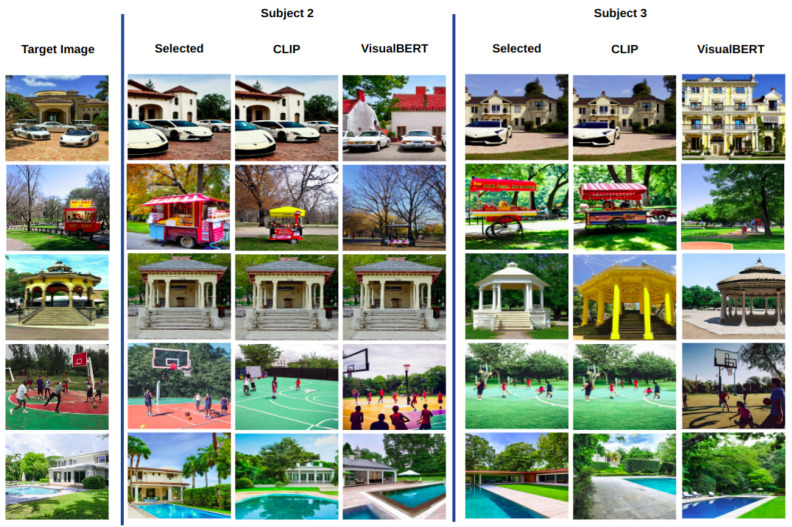
Same comparison as in Figure 5 but without showing the prompts and scores.

We ask the reader to remember that in each case, the best image was drawn from 100 images per target image. Note that 4 out of 5 images evaluated with CLIP coincide with those selected by Subject 1. In the case of VisualBERT, there is no coincidence, yet the best images keep a resemblance in semantic terms. For completeness, in Figure 6, we also show the best images for Subjects 2 and 3, this time without the prompts. Again, CLIP draws more coincidences with the images selected by both users. In contrast, VisualBERT coincides only one time for Subject 2 on the third target image, the kiosk image. Once more, the images may not coincide with those selected by the users, but the resemblance with the target image in terms of objects, shapes, and colours in the scene is uncanny.

### 4.2. Textual Descriptions vs. Automatically Generated Text

**Figure 7:**
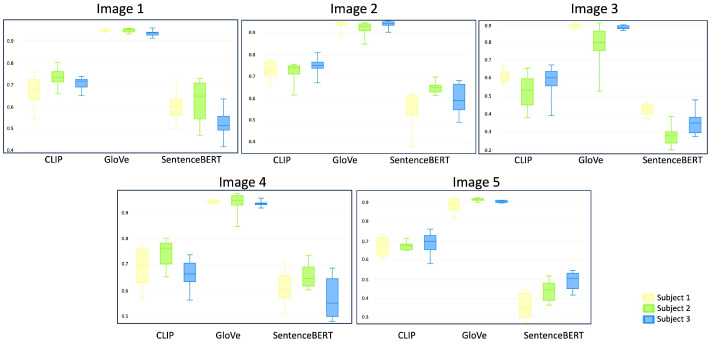
Box plots summarising the similarities obtained by comparing the textual descriptions generated by the subjects and the target texts.

One of the intuitive strategies could involve comparing text-to-text information. Therefore, we decided to evaluate the similarity scores obtained by comparing the target images’ textual descriptions, obtained through the image captioning model, against the textual descriptions or prompts provided by the subjects for image generation. This approach involves the utilisation of GloVe, SentenceBERT, and CLIP to represent textual information in a vectorial space and subsequently gauge the similarity scores. Figure 7 shows the obtained results summarised by means of boxplots regarding the cosine similarity between the automatic and human-generated textual descriptions for each target image. As can be observed, the highest similarity values are obtained by the GloVe representation for all images and all users, while the lowest were with SentenceBERT. Overall, the similarities obtained are lower than the ones obtained when using visual information. Then, making a direct comparison between the use of both modalities is not a trivial issue. It is important to highlight that since we are assuming the textual descriptions generated by image captioning are the ”target texts”, all the comparisons performed must be carefully interpreted in the sense that the captions depend entirely on the pre-trained model exploited. For instance, we noticed that these textual descriptions are shorter than most of the user-generated ones, which mostly attempt to consider particular details of the target images. We consider that exploring the use of textual-based comparison is a topic that deserves to be further investigated.

In most cases, the similarities obtained for each image are comparable between the textual descriptions. However, some salient differences can be observed in some cases. For example, let us consider Image 3 and Subject 2. The textual descriptions generated by the user for this image are particularly short (one of them has only 3 words). The similarity values obtained are very dispersed for all text representations; the ones obtained with GloVe are notably diverse, while the values obtained by Subject 1 and Subject 3 for the same image and text representation are more similar between them. We hypothesise that this could be because Subject 2 included some out-of-vocabulary words in their textual descriptions.

**Figure 8:**
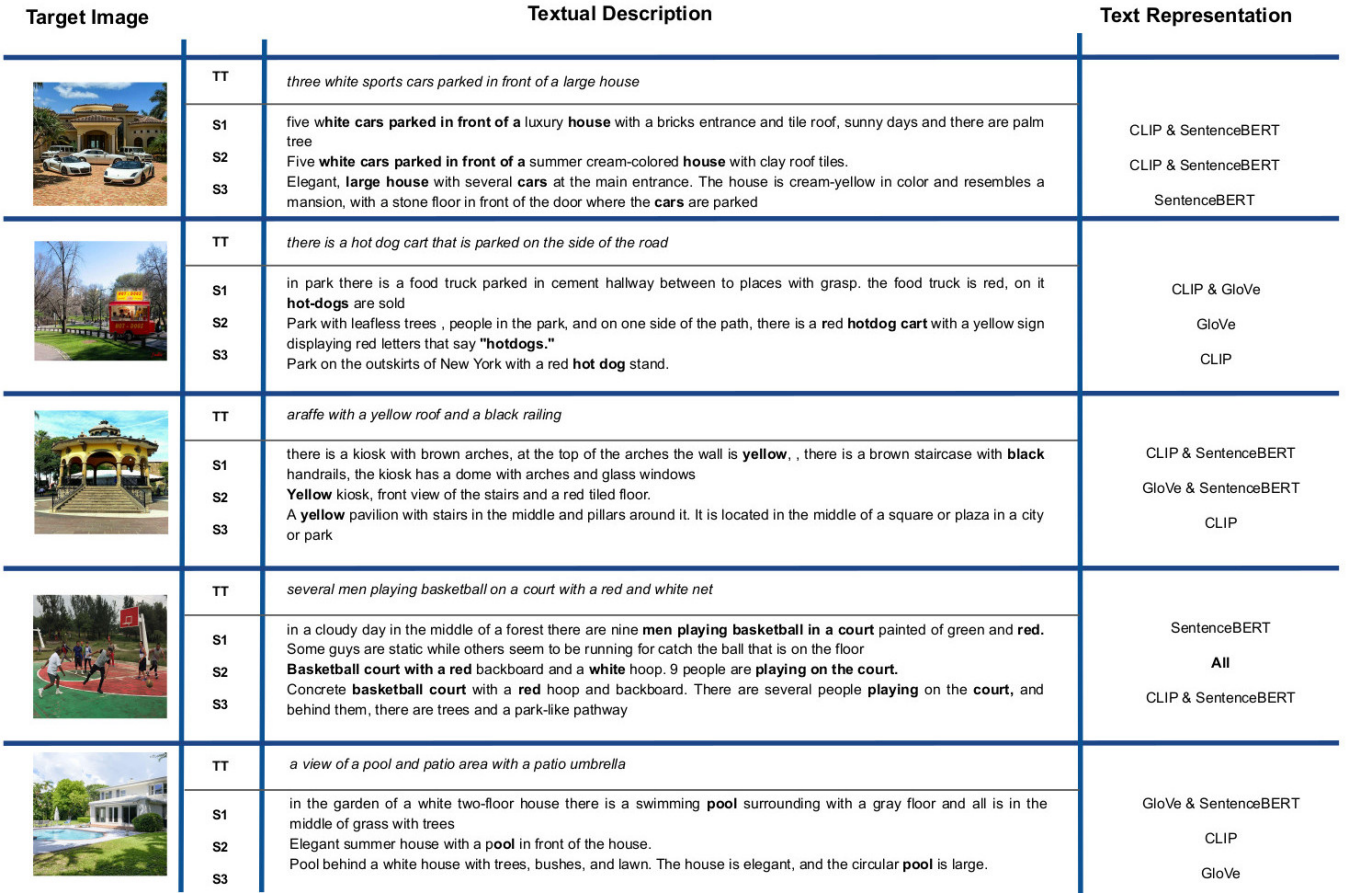
Examples of human-generated descriptions for each target image. The automatic descriptions are also included.

In a similar fashion to the first strategy, we decided to analyse which of the human-generated textual descriptions are more similar to the “target texts”. To do so, we identified which of the textual descriptions obtained the highest similarity with respect to the target text and then determined whether or not this greater similarity similar is the same for all text representations. Figure 8 shows some human-generated textual descriptions together with their corresponding “target text” (denoted as ‘TT’). The last column indicates which of the text representation methods was used in each case. When two models appear, this is because both textual descriptions achieved the highest similarity value. In the rest of the cases, there is no agreement between the text representations. It is important to note that only in one case is there a full agreement with all text representations.

For each target image and its corresponding textual descriptions, we highlight in bold those main concepts that appear in both kinds of descriptions. As can be observed, each human-generated text contains at least one keyword in common with the TT. For Images 1 and 4, we identified the largest sequences of words, while for the rest of the TT, only one word was common in the textual descriptions. A very particular case is once again Image 3, in which a very uncommon word (“araffe”) was obtained from the image captioning model. It is very likely that such a word would not be used by the subjects to describe the scene of interest. The use of this kind of non-common term to refer to an important object in the scene is another particular aspect that needs to be further explored.

### 4.3. Enhancement of Users’ Textual Descriptions

As indicated in Section 3, we used ChatGTP to generate an enhanced version of each textual description out of the textual descriptions provided by each participant for each target image. In doing this, we aimed to evaluate whether this improved prompt would help to generate an image more similar to the target image. To this end, we use the participants’ prompts to feed ChatGPT, as follows:

- “I have 10 descriptions of a place: [here the list of human-created prompts were added] for each one of the 10 descriptions, could you tell me the average number of words?”; and
- “ok, then given these 10 descriptions, could you mix them to generate the best description whose number of words is around [NUM] words? (where NUM was replaced by the average length of the prompts generated by each subject)”.

As output, we obtained a new prompt for each target image per subject. As before, we passed these prompts to Stable Diffusion to generate 10 images per enhanced prompt. The generated images were evaluated using CLIP and VisualBERT as in Section 4.1. Note that we used the enhanced prompts with each generated image for the VisualBERT embedding.

**Figure 9:**
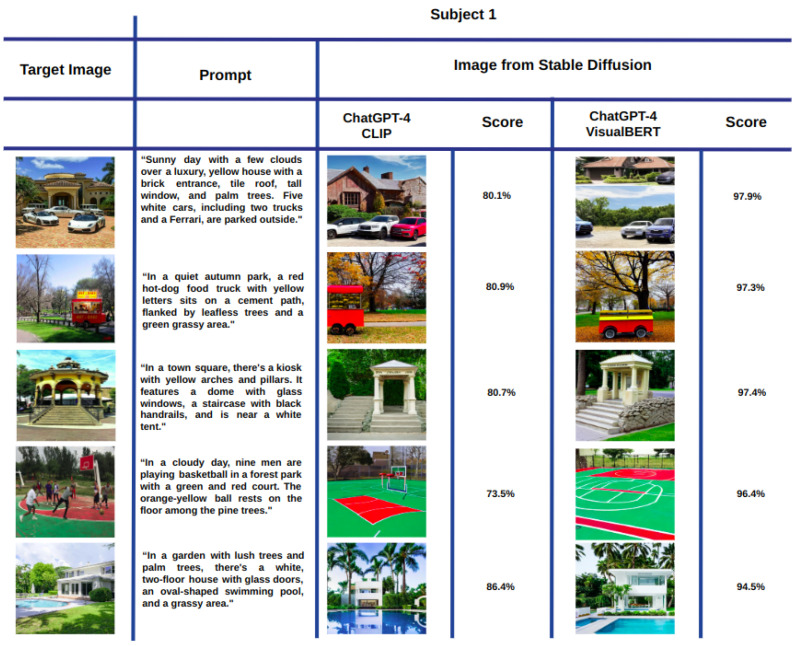
Best images generated with the enhanced prompt of Subject 1 using ChatGPT, including their score, when using CLIP and VisualBERT.

**Figure 10:**
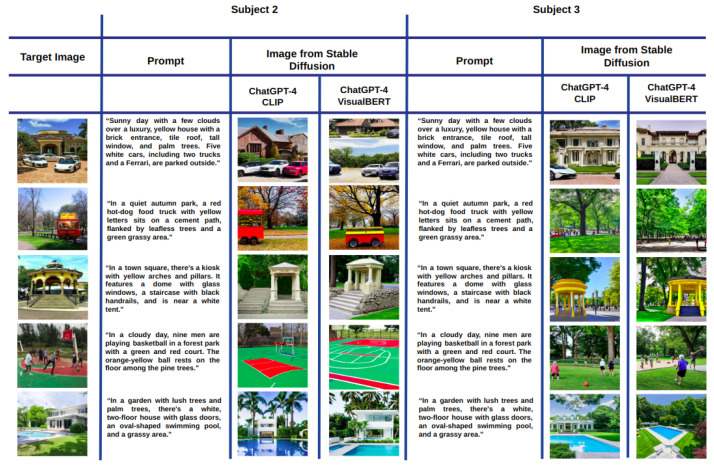
Best images generated with the enhanced prompt of Subject 1 and Subject 2 using ChatGPT when using CLIP and VisualBERT.

Therefore, the images with the highest score (using CLIP and VisualBERT) for each target with the summary prompt are shown in Figure 9 for Subject 1. Note that once more, the best images drawn according to the cosine score, either using CLIP or VisualBERT, have a high score considering the score distributions shown in Figure 4, but also in semantic terms, the generated images contain similar objects to those found in the target images. However, we noticed that these images were not better than those obtained when using the variations of prompts. This can also be noted in the images drawn for Subject 2 and Subject 3, shown in Figure 10. This could indicate that when it comes to searching space, it would be better to generate a wider set of images from variations of prompts rather than using a particular one that could limit the text-to-image generative model.

### 4.4. Discussion

Table 1 shows the highest scores obtained with both CLIP and VisualBERT for each target image and per subject. Remember that, for a target image, the score corresponds to the highest score out of the 100 scores measured with the corresponding embeddings, that is, 100 images generated from 10 prompts for each image, per participant. The values in the table are useful to appreciate the maximum scores obtained with both types of embeddings, and by looking at the images associated with these scores, we can establish that these high values indicate that the images do resemble the target image in semantic terms. Therefore, this methodology could be used to visually recognise a target scene using only a quantitative textual description. Furthermore, these sets of scores in Table 1 could also help to determine the threshold that determines whether the target place is recognised or not.

Additionally, we present the best scores for enhanced textual descriptions in Table 1, obtained for each target image based on user and embedding type. We observed a consistent trend in score values, where the most similar images closely align. Specifically, images drawn from the pool of generated images using enhanced prompts exhibit both visual and semantic similarity to the target images.

However, it is noteworthy that, this time, none of the images coincided with those selected by the subjects. This suggests that, qualitatively, the enhanced prompts may not have contributed to generating better images. Nevertheless, this discrepancy could be attributed to the smaller pool of generated images compared to previous experiments.

Thus, the key insight is that, for the effective use of generated images from text in recognising unknown target scenes, one must be able to create a diverse pool of generated images. It is evident that CLIP or VisualBERT can assist in determining which image will score better when it becomes visually and semantically similar to the target image.

In summary, our results indicate that among the three proposed strategies—image generation, text generation, and text enhancement—the most promising one is centred on image generation using textual descriptions. We evaluated the use of text generation models against human-generated textual descriptions to illustrate that, even though this approach might demand less computational effort, it proves to be less effective compared to utilising generated images. Lastly, we introduce text enhancement using ChatGPT, where we have discovered that a more varied textual description yields better results than a concise one.

## 5. Conclusions

This work has been motivated by the last-mile delivery problem, in which a courier has to find a place they have probably never been before. In anticipation of this issue, it has become a common practice for delivery companies, in particular for those in e-commerce, to request a textual description of the delivery destination, hoping that such a description could aid the courier in visualising what the target destination looks like. Therefore, we have explored the use of generative models to develop a methodology that enables an artificial agent, such as a delivery drone or service robot, to mimic the process of “imagining” an unknown target scene by means of a textual description only. To this end, we have explored the use of a text-to-image generative model, image captioning, as well as multi-modal vision-and-language models such as CLIP and VisualBERT for text and image representation via numerical embeddings. Our experiments show that a generative model such as Stable Diffusion can be used to generate images visually and semantically similar to target images of unknown scenes in both qualitative and quantitative terms with no prior information or cues about these images.

For our future work, we will investigate novel generative methods that could be run in real-time, a crucial aspect for aerial and service robots. This also calls for a deeper study of the text modality, which could be faster to process to rule out dissimilar images, leaving the final decision to the image generation-based method.

## Figures and Tables

**Table 1:** Best scores for images generated through human-generated descriptions and ChatGPT prompts obtained using CLIP and VisualBERT.

Target Image	Best Score											
	**CLIP**			**VisualBERT**			**CLIP GPT-4**			**VisualBERT GPT-4**		
	**S1**	**S2**	**S3**	**S1**	**S2**	**S3**	**S1**	**S2**	**S3**	**S1**	**S2**	**S3**
1	89.84	84.08	84.77	98.21	98.74	94.68	84.66	80.08	76.94	99.48	99.71	99.49
2	82.86	83.35	83.99	97.78	98.27	98.35	83.78	84.57	88.33	99.44	98.94	99.4
3	84.08	80.27	81.20	97.45	98.38	97.35	83.48	89.37	86.39	99.6	99.42	99.66
4	79.00	77.69	79.30	96.9	98.33	98.06	85.68	90.06	83.18	98.25	98.07	99.43
5	86.28	89.84	90.62	97.13	98.57	93.71	90.98	92.35	90.68	98.37	98.39	99.33

## Data Availability

Target and generated images as much as textual descriptions will be available upon request.
